# A multimodal data-set of a unidirectional glass fibre reinforced polymer composite

**DOI:** 10.1016/j.dib.2018.04.039

**Published:** 2018-04-14

**Authors:** Monica J. Emerson, Vedrana A. Dahl, Knut Conradsen, Lars P. Mikkelsen, Anders B. Dahl

**Affiliations:** a Department of Applied Mathematics and Computer Science, Technical University of Denmark, Denmark; b Department of Wind Energy, Technical University of Denmark, Denmark

**Keywords:** Geometrical characterisation, Polymer-matrix composites (PMCs), Volumetric fibre segmentation, Automated fibre tracking, X-ray imaging, Microscopy, Non-destructive testing

## Abstract

A unidirectional (UD) glass fibre reinforced polymer (GFRP) composite was scanned at varying resolutions in the micro-scale with several imaging modalities. All six scans capture the same region of the sample, containing well-aligned fibres inside a UD load-carrying bundle. Two scans of the cross-sectional surface of the bundle were acquired at a high resolution, by means of scanning electron microscopy (SEM) and optical microscopy (OM), and four volumetric scans were acquired through X-ray computed tomography (CT) at different resolutions. Individual fibres can be resolved from these scans to investigate the micro-structure of the UD bundle. The data is hosted at https://doi.org/10.5281/zenodo.1195879 and it was used in Emerson et al. (2018) [1] to demonstrate that precise and representative characterisations of fibre geometry are possible with relatively low X-ray CT resolutions if the analysis method is robust to image quality.

**Specifications Table**

**Table:** 

Subject area	*Physics*
More specific subject area	*Fibre composites, micro-structure characterisation, geometry of individual fibres*
Type of data	*Image (X-ray and microscopy)*
How data was acquired	– *Optical microscopy (OM): Leica DMI5000 M.* – *Scanning Electron Microscopy (SEM): Carl Zeiss AG SUPRA 35.* – *Laboratory X-ray CT (XCT): ZEISS Xradia 520 Versa.* – *Synchrotron X-ray CT (SRCT): ID19 beamline from the European Synchrotron Radiation Facility (ESRF).*
Data format	*Raw (microscopy), reconstructed (X-ray CT)*
Experimental factors	– *The surface of the sample was polished before acquiring the OM scan.* – *To acquire the SEM scan the surface of the sample was made conductive by adding a coating of gold.*
Experimental features	*Two surface and four volumetric scans capturing the same region of the specimen with pixel sizes ranging from 0.18 μm to 2.81 μm.*
Data source location	*Roskilde, Denmark and Grenoble, France.*
Data accessibility	*The data can be downloaded from:*https://doi.org/10.5281/zenodo.1195879
Related research article	*The data-sets presented in this paper have been used in*[1]*to demonstrate the precision of X-ray CT for characterising fibre geometry in unidirectional composites at the micro-scale. In*[1]*we also demonstrate that high-precision measurements can be obtained from low-resolution X-ray CT scans if coupled with analysis methods that are robust to image resolution, such as the individual fibre segmentation in*[2]*.Obtaining precise measurements from low-resolution X-ray CT scans will facilitate the analysis of larger volumes, enabling quantifications that are more representative than what has been obtained in other studies. As shown in*[1]*, the geometry of individual fibres can be characterised with high precision in a fast and reliable manner using laboratory micro-CT scanners.*

**Value of the Data**

• This data can be employed to test methods for individual segmentation of fibres. By analysing the area of great overlap across scans, it is possible to assess the imaging modalities to which a segmentation method is applicable. Additionally, it is possible to investigate the robustness of the segmentation method to image pixellation and determine whether precise measurements can be obtained from low-resolution scans that capture fields of view containing a representative number of fibres.
• This data can be used to quantify aspects of the fibre geometry, such as individual fibre diameters [1] and orientations [2], local fibre volume fraction or fibre contact points. The quantification of fibre geometry obtained from precise measurements can provide insights into the fibre and composite's manufacturing processes. Real samples differ from the design criteria and it is of interest to study the variability of the fibre geometry in 3D, as it strongly affects the mechanical performance of the composite.
• The fibre geometry measurements obtained from these data-sets can also be employed for generating two- and three-dimensional micro-mechanical models with the purpose of simulating the behaviour of the real sample under load [3].

## 1 Data

**Fig. 1:**
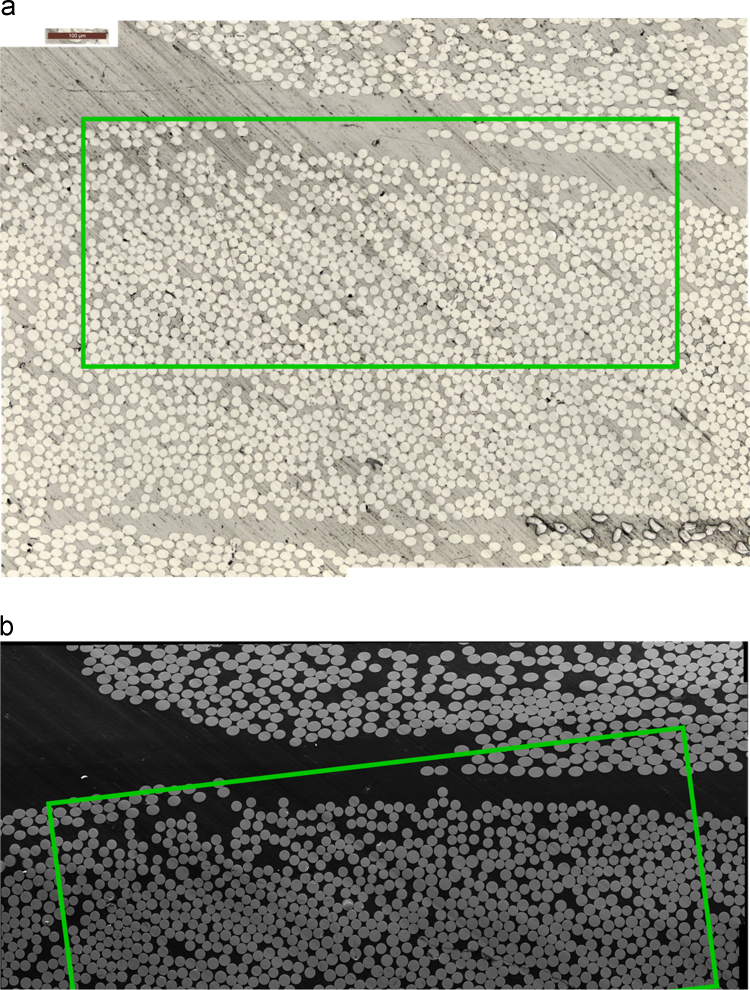
Surface scans with the sample area of great overlap across scans marked in green. In (a) the image acquired through optical microscopy and in (b) through scanning electron microscopy.

**Fig. 2:**
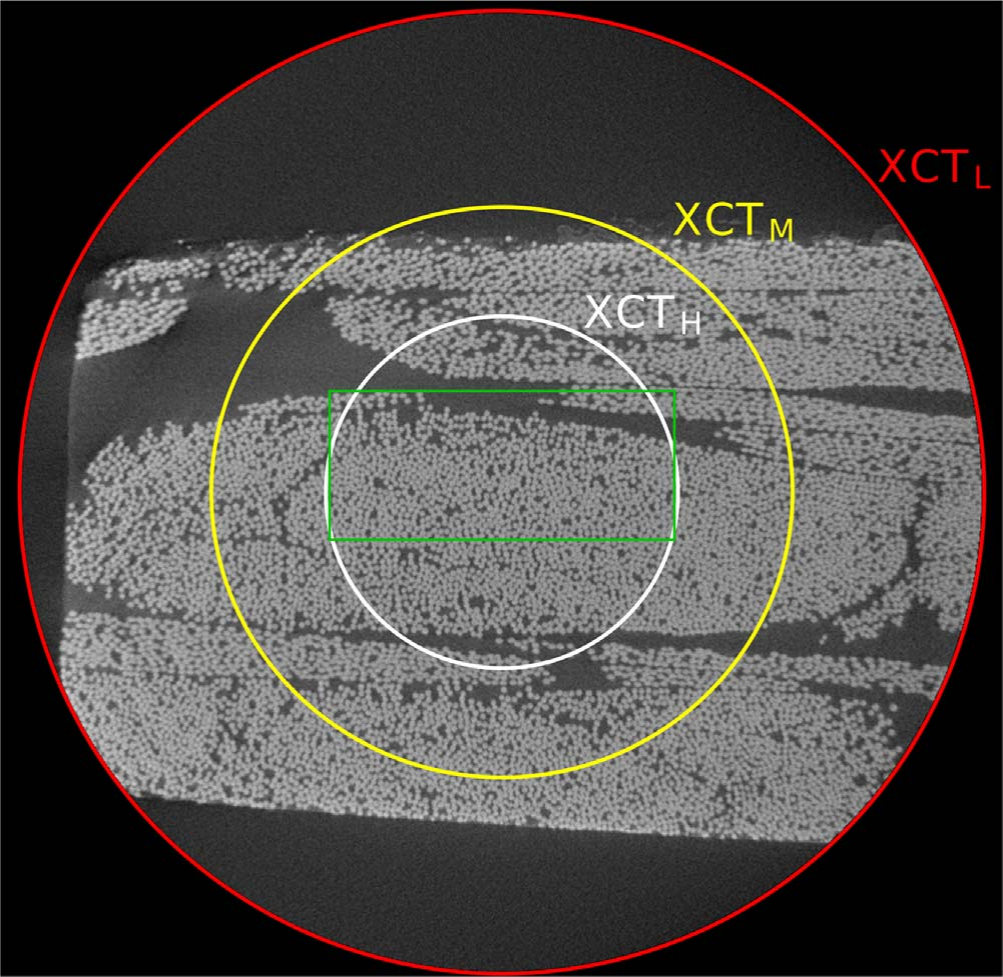
X-ray CT cross-sectional slice for the low-resolution scan, illustrating the decrease in field of view for scans with increased spatial resolution. The sample area of great overlap across scans has been marked in *green* and the fields of view for the low- (XCT_L_), mid- (XCT_M_) and high- (XCT_H_) resolution scans have been marked in *red*, *yellow* and *white* respectively.

**Fig. 3:**
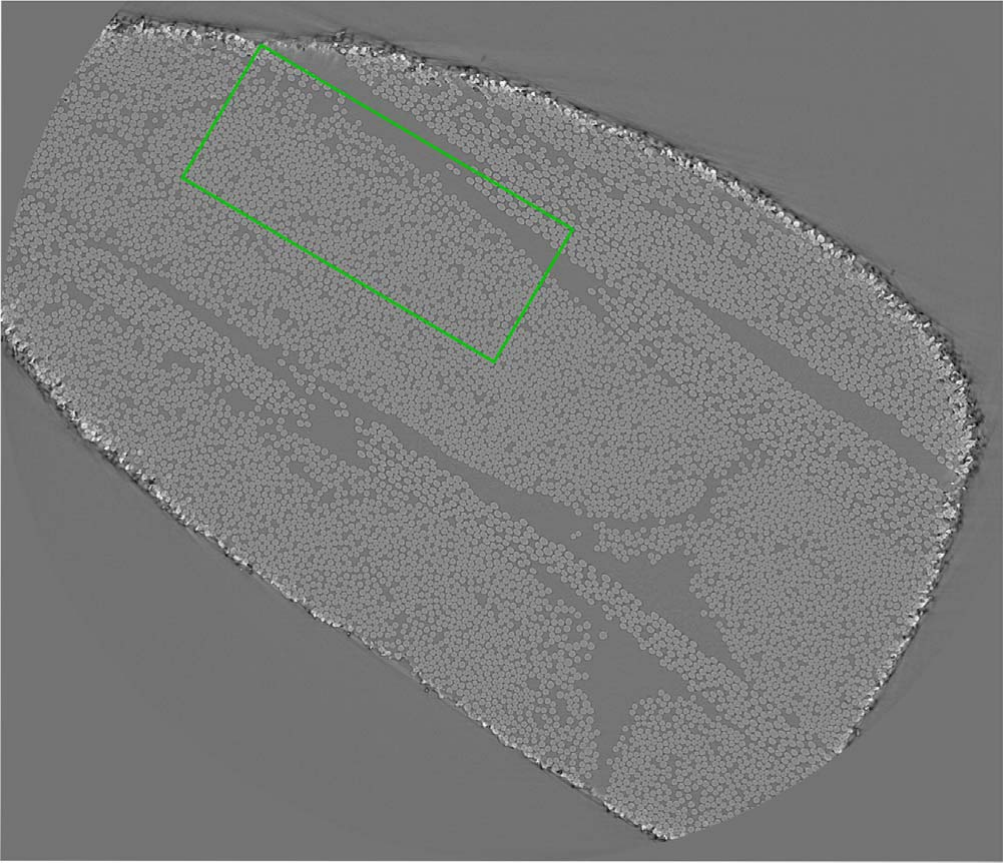
X-ray CT cross-sectional slice for the synchrotron scan with the sample area of great overlap between all six scans indicated in *green*.

The data presented in this article consists of six scans. Two surface scans acquired with optical microscopy and scanning electron microscopy (see Fig. 1) and four volumetric scans acquired by means of X-ray computed tomography (CT) at a laboratory (three different resolutions, see Fig. 2) and a synchrotron source (see Fig. 3).

As can be seen in Fig. 1, Fig. 2, Fig. 3, the resolutions and fields of view (FoV) vary for all six data-sets. The pixel sizes and FoV are reported later on in this article. There is an area of the sample captured approximately by all six scans. This area of great overlap is marked in *green* in Fig. 1, Fig. 2, Fig. 3.

The optical microscopy scan is given under the folder “OM” as one “.tif” image whereas the scanning electron microscopy scan is given under the folder “SEM” as a set of 49 “.tif” images. These 49 images were fused using ImageJ to obtain the image in Fig. 1b, which is provided as a “.jpg” image inside the folder “SEM”.

As to the three-dimensional X-ray scans, the reconstructed CT volumes are given as a series of “.tif” cross-sectional slices. We are sharing the full volumes for the scans acquired at the laboratory scanner, along with the relevant scan settings (labelled “info1” and “info2”). The three volumes are under the folders named “XCT_L”, “XCT_M” and “XCT_H” corresponding to the three spatial resolutions: low, mid and high. Fig. 2 shows the cross-sectional slice closest to the top surface of the sample for the low-resolution data-set, where the FoV for the higher resolution scans has been indicated over the cross-sectional image.

The fourth X-ray CT scan was acquired at ID19, beamline of the European Synchrotron Radiation Facility (ESRF). This scan is higher in resolution than the laboratory scans and also covers a larger region of the sample, see Fig. 3 for the cross-sectional slice that is closest to the imaged surface. While the scans acquired at the laboratory scanner occupied under 1.3 GB, the full high-resolution synchrotron scan is over 100 GB. Thus, we have decided to share only 61 full-resolution cross-sectional slices, covering a depth of 0.6 mm from the surface of the sample.

## 2 Experimental design, materials, and methods

The scanned unidirectional (UD) glass fibre composite is a non-crimp fabric commonly used in the load-carrying parts of wind turbine blades, for details on this type of composite see [4]. The imaged sample of cross-sectional size 2 mm × 2 mm consists of UD fibre bundles stitched on backing bundles angled 45°, −45° and 90° with respect to the UD (0°) bundles. For more details and illustrations see [1], [4].

The sample was scanned under no load. The surface of the UD bundle was imaged through optical and scanning electron microscopy and the internal micro-structure was imaged using X-ray CT. Three scans were acquired at a laboratory source at three different resolutions and a fourth scan was acquired at a synchrotron facility at a higher resolution. The pixel sizes for the six scans are reported in Table 1 and details for the four different imaging sources are given in the following.

**Table 1:** Pixel sizes (as reported by the instruments) and fields of view for the six scans. For the 3D scans the depth is also provided. NR where not relevant.

Data-set	Pixel size [μm]	Fields of View (FoV)	Depth
SEM	0.19	0.56 mm × 1.17 mm^a^	NR
OM	0.29	0.61 mm × 0.82 mm	NR
SRCT	0.65	2.82 mm × 2.82 mm	0.63 mm (just 61 slices)
XCT_H_	1.04	1.05 mm × 1.05 mm	0.65 mm
XCT_M_	1.69	1.71 mm × 1.71 mm	0.96 mm
XCT_L_	2.81	2.84 mm × 2.84 mm	1.53 mm

a after stitching 49 scans.

The optical microscopy scan was acquired using the objective ×20 of the Inverted Research Microscope for Materials Testing Leica DMI5000 M. Before taking the OM image, the sample was polished using a Tegramin machine from Struers.

The scanning electron microscopy image was acquired using the Carl Zeiss AG - SUPRA 35 with an in-lens SE2 secondary electron detector, an acceleration voltage of 15 kV, a working distance of 9.1 mm and a magnification of ×2160. Before acquiring the scan, the surface of the sample was coated with gold using a BALTEC SCD 005 sputter coater. A sputtering current of 30 mA and a sputtering time of 76 s were set in order to obtain a 10 nm thick layer of gold.

Three X-ray CT scans were acquired with the laboratory micro-focus X-ray CT system Zeiss Xradia 520 Versa. The settings for the three scans are reported in Table 2.

**Table 2:** X-ray CT scanner settings.

Data-set	XCT_L_	XCT_M_	XCT_H_
Optical magnification	4.01(4×)	4.01(4×)	4.01(4×)
Source to sample distance	10 mm	10 mm	10 mm
Detector to sample distance	14 mm	30 mm	55 mm
Exposure time (per projection)	0.5 s	1 s	4.5 s
Accelerating voltage	80 keV	80 keV	80 keV
Power	6.99 W	6.99 W	6.99 W
Number of projections	4201	3201	4201

The synchrotron X-ray CT scan was acquired at the ID19 beamline of the European Synchrotron Radiation Facilty (ESRF) during the 16-bunch top-up mode. The synchrotron radiation was produced with the undulator U13, which creates a spectrum with a narrow peak in the energy of 26.3 keV. The detector consisted of a PCO.edge 5.5 camera with an optical magnification of 10× and a GGG10 scintillator. The detector was placed at a distance of 13 mm from the sample, which resulted in a voxel size of 0.65 mm. The sample was rotated 360° with the centre of rotation placed on the side of the projection, so as to double the horizontal field of view. The number of projections acquired was 4608 and the exposure time 0.1 s.
