# Supplementary material for: A multimodal data-set of a unidirectional glass fibre reinforced polymer composite
Source: Data Brief. 2018 Apr 14;18:1388–93. doi: 10.1016/j.dib.2018.04.039 (PMC6060273; doi:10.1016/j.dib.2018.04.039)
Supplement: Supplementary file 1 — Supplementary material [file mmc1.pdf]

Dear Editor,

The authors declare no competing financial interests.

In behalf of all authors,

Monica Jane Emerson
5th April 2018
